# Prolonged response to first-line erlotinib for advanced lung adenocarcinoma

**DOI:** 10.1186/1756-9966-27-59

**Published:** 2008-11-04

**Authors:** Michael Copeman

**Affiliations:** 1 Northern Beaches Cancer Service, Sydney, Australia

## Abstract

A 58-year-old, non-smoking female of Philippine origin presented with painful thoracic and neck nodal relapse of lung adenocarcinoma almost 5 years after left pneumonectomy for stage II non-small-cell lung cancer. She refused conventional chemotherapy or radiation because of toxicity concerns, but agreed to oral erlotinib 150 mg/day. Within weeks, her pain was well controlled, with softening of palpable neck nodes. Repeat scans after 7 months on erlotinib showed partial response of thoracic disease and nodal metastases. This response was maintained for 11 months on erlotinib, with symptomatic progression at the original sites of relapse by 15 months. Erlotinib was well tolerated, with grade 2–3 rash, and grade 1 dry cough and diarrhoea being the only significant toxicities. Importantly, the patient was able to maintain daily activities throughout erlotinib therapy.

## Background

For patients diagnosed with non-small cell lung cancer (NSCLC), platinum-based chemotherapy is frequently used as a first-line treatment [1,2]. Most patients will eventually exhibit signs of disease progression following first-line chemotherapy, and a second line of therapy may be required to control tumour growth. In a phase III trial, the epidermal growth factor receptor (EGFR) inhibitor erlotinib has been shown to significantly prolong survival, delay symptom deterioration, and provide quality-of-life benefits for patients who experienced disease progression following first-line chemotherapy [3,4].

Due to the cytotoxic effects of chemotherapy, some patients may be considered unsuitable for chemotherapy or may decline chemotherapy as a first-line treatment. In these circumstances, erlotinib may be used as an alternative and less toxic first-line therapy. This report describes the case of a NSCLC patient who had a prolonged radiological and symptomatic response to first-line erlotinib therapy.

## Case report

A 58-year old non-smoking female, of Philippine origin but domiciled in Australia, presented in December 2000 with cough and wheeze that was not responsive to inhaled beta-agonists. Chest radiography followed by computerised tomography (CT) showed a stage II left perihilar lung tumour, which was confirmed as NSCLC with adenocarcinoma histology by fine needle biopsy. The patient was otherwise well, but had a past history of chronic endometriosis. Her non-smoking mother died of lung cancer at the age of 72, but there was no other family history of cancer. The primary lung tumour and hilar nodes were successfully removed by left pneumonectomy in January 2001. The patient declined adjuvant chemotherapy or radiation, because of concerns about toxicity.

In August 2005 the patient returned to her respiratory physician with weight loss, enlarged left neck lymph nodes, and upper left neck and thoracic pains not controlled by paracetamol and codeine. CT scan showed adenocarcinoma (confirmed on needle biopsy) in the left cervical nodes (> 2.5 cm), a 3.3 cm mass at the left pneumonectomy stump, a 1.2 cm right middle lobe lung lesion, and possible liver metastases up to 1.8 cm in size. Transdermal fentanyl patches were commenced for pain relief but the patient again declined cytotoxic chemotherapy or radiation because of toxicity concerns. Erlotinib was, therefore, recommended as an alternative to chemotherapy.

The patient started oral erlotinib 150 mg/day in September 2005, and within weeks noticed softening of the left cervical nodes, and a decrease in pain, beyond that achieved with opiates. She developed the facial and anterior neck rash typical of EGFR inhibitors, with progression to a grade 3 reaction. The rash did not resolve with topical emollients or corticosteroids, but the patient decided to continue therapy and the rash spontaneously resolved to grade 2 in the next few months. Grade 1 dry cough and mild diarrhoea were also noted, which both persisted throughout. Repeat CT scan in January 2006 showed resolution of the liver lesions and a decrease in both thoracic lesions; there was no change in the mediastinal or left neck nodes. By May 2006, the cervical nodes had decreased below 1 cm diameter, the left pneumonectomy stump mass was 1.6 cm, and there were no new lesions, consistent with a partial response.

In August 2006, after 11 months of erlotinib therapy, there was radiographic progression, with an increase in the left stump mass to 1.8 cm and the right middle lobe lesion to 1.4 cm. However, there were no new lesions, and the patient remained on erlotinib with an absence of new symptoms. By December 2006, the left cervical nodes had enlarged further, and the patient had increased pain in her left neck and upper thorax. At this time, erlotinib was discontinued and the patient agreed to start conventional chemotherapy with paclitaxel and carboplatin, which produced a partial response and symptomatic benefit. Since January 2008, the patient has been receiving palliative care, with the latest follow up in October 2008.

## Discussion

Chemotherapy options for advanced NSCLC are associated with haematological toxicity that may reduce patients' quality of life (QoL), or necessitate hospitalisation. As a consequence, many patients are either unsuitable or, as in the case described here, unwilling to receive chemotherapy. Erlotinib, a potent inhibitor of EGFR tyrosine-kinase activity has similar efficacy to second-line chemotherapy options but a more favourable tolerability profile [5]. In the BR.21 phase III trial, erlotinib provided a significant survival benefit over placebo in patients with advanced NSCLC who had previously received chemotherapy (hazard ratio 0.70, 95% confidence intervals 0.58–0.85, p < 0.001) [3], leading to regulatory approvals in this setting. Erlotinib is currently under investigation as a front-line treatment for advanced NSCLC, and several phase II studies of first-line erlotinib monotherapy have been reported, with promising results observed in both selected and unselected patient populations [6-9]. Ongoing studies also seek to determine which patients are most likely to obtain a clinical benefit from first-line erlotinib therapy. In the BR.21 study, female gender, adenocarcinoma histology, never smoking status, and Asian ethnicity were associated with a significantly greater likelihood of response to erlotinib. The patient described in this report had all of these characteristics and obtained a prolonged radiographic and symptomatic response to first-line erlotinib. However, these demographic and clinical characteristics do not provide criteria for selecting patients to receive erlotinib; in the BR.21 study, survival benefits were observed in almost all patient subgroups studied, including in male former/current smokers with squamous-cell carcinoma, a group that would be expected to have a low tumour response rate [3,10]. Tumour molecular markers, particularly EGFR protein expression, *EGFR *gene copy number and the presence of mutations in the EGFR tyrosine-kinase domain, have also been studied to determine which patients are most likely to benefit from erlotinib therapy [11]. However, none of these factors were significantly associated with survival on erlotinib therapy in the BR.21 trial, and further investigation is required [12,13]. Therefore, at present there are no reliable criteria for selecting patients to receive erlotinib therapy.

Rash and diarrhoea were the only significant toxicities observed in the current case, and are the most common toxicities associated with erlotinib. These toxicities are generally mild or moderate and easily managed [3,14]. Furthermore, as seen in this case, rash often resolves to a lower grade without any need for dose modification. In addition to having a favourable tolerability profile, erlotinib provides significant symptom and QoL benefits [3,4]. The patient described here obtained 15 months of relief from cancer-related pain on erlotinib therapy, while maintaining most daily activity. Such improvements in symptoms and QoL are highly valued by patients, even in the absence of survival benefits [15].

## Conclusion

In conclusion, this report describes an impressive radiological and symptomatic response in a patient receiving first-line erlotinib for advanced NSCLC. Erlotinib is, therefore, an attractive option for NSCLC therapy.

## Consent

Written informed consent was obtained from the patient for publication of this case report. A copy of the written consent is available for review by the Editor-in-Chief of this journal.

## Competing interests

MC has received support from Roche, manufacturer of Tarceva (erlotinib), to attend an overseas medical conference in 2007.
